# Current and novel approaches to vaccine development against tuberculosis

**DOI:** 10.3389/fcimb.2012.00154

**Published:** 2012-12-06

**Authors:** Mark J. Cayabyab, Lilia Macovei, Antonio Campos-Neto

**Affiliations:** ^1^Forsyth InstituteCambridge, MA, USA; ^2^Harvard School of Dental MedicineBoston, MA, USA

**Keywords:** tuberculosis, *Mycobacterium tuberculosis*, vaccine, antigen, adjuvant, recombinant protein, delivery system

## Abstract

Antibiotics and vaccines are the two most successful medical countermeasures that humans have created against a number of pathogens. However a select few e.g., *Mycobacterium tuberculosis (Mtb)*, the causative agent of tuberculosis (TB) have evaded eradication by vaccines and therapeutic approaches. TB is a global public health problem that kills 1.4 million people per year. The past decade has seen significant progress in developing new vaccine candidates, but the most fundamental questions in understanding disease progression and protective host responses that are responsible for controlling *Mtb* infection still remain poorly resolved. Current TB treatment requires intense chemotherapy with several antimicrobials, while the only approved vaccine is the classical viable whole-cell based Bacille-Calmette-Guerin (BCG) that protects children from severe forms of TB, but fails to protect adults. Taken together, there is a growing need to conduct basic and applied research to develop novel vaccine strategies against TB. This review is focused on the discussion surrounding current strategies and innovations being explored to discover new protective antigens, adjuvants, and delivery systems in the hopes of creating an efficacious TB vaccine.

## Tuberculosis disease and epidemiology

Tuberculosis (TB) has been one of the major causes of morbidity and mortality worldwide for centuries. TB takes a devastating toll on human lives with 2.2 billion people, representing one-third of the world's population, currently infected with the causative agent, *Mycobacterium tuberculosis (Mtb)*, and an alarming 1.4 million deaths each year (WHO, 2012). TB is a complicated disease with multiple infectious sites and a wide range of clinical disease manifestations. Approximately 70% of *Mtb*-exposed individuals will clear the bacteria, while the remaining 30% will get infected. In about 90% of those infected, *Mtb* either gets controlled by the immune response or remains viable but physiologically inactive or dormant in the host. People infected with dormant *Mtb* are diagnosed as having latent TB or non-clinical TB infection (LTBI). It is thought that 5–10% of LTBI cases progresses to active disease. Reactivation of latent TB may occur during the chronic stage of infection, possibly due to the change of the host's immune response or because of exogenous reinfection with *Mtb*.

Immunodeficiency in individuals co-infected with the human immunodeficiency virus type 1 (HIV-1) causes a breach in host containment of *Mtb* resulting in increased incidence of reactivation of TB. With the spread of human immunodeficiency virus (HIV) and dramatic increase in the number of cases of multidrug-resistant (MDR-TB) and extremely drug-resistant (XDR-TB) infections, the WHO in the early 1990's declared TB as a re-emerging infectious disease. WHO estimates that there are more than 8.7 million new cases of active TB each year (WHO, 2012) and about 2.6 million new cases of HIV infection with 1.8 million AIDS-related deaths per year (UNAIDS, 2010). Alarmingly, about 14 million individuals worldwide are estimated to be dually infected with TB-HIV (Getahun et al., 2010). TB is the largest cause of death in AIDS patients, (~29%), most of which (99%) occur in developing countries (Pawlowski et al., 2012). In HIV/TB co-infection, both pathogens combat the host's immune responses through various mechanisms. HIV depletes CD4+ T cells of the lamina propria of the gastrointestinal tract (Clayton et al., 1992; Brenchley and Douek, 2008) resulting in T cell dysfunction in cytokine production and cytotoxic activity, and therefore compromising the immune response. HIV/TB co-infection dramatically increases the risk of latent TB reactivation by 20- to 37-fold, and is therefore the most powerful risk factor for latent TB reactivation (Getahun et al., 2010).

Antimicrobial therapy with various regimens is used to treat TB. However, their inadequate and inappropriate use has triggered an increase in MDR-, XDR-TB, and total drug-resistant (TDR-TB) cases worldwide, adding to the challenges of TB eradication and healthcare cost (Caminero, 2010). Based on drug resistance data from 114 countries and two-regions of China, it was estimated that ~500,000 MDR-TB cases emerged in 2006, representing 4.8% of all cases of TB (WHO, 2007). While drug-sensitive strains of *Mtb* in immunocompetent patients are treated for at least 6 months with multiple antibiotic regimens to achieve efficient clearing of the mycobacteria by the host, in MDR-TB or XDR-TB cases second-line drugs are used, with a higher risk of adverse effects and lower potency, raising the costs of treatment for patients carrying MDR strain dramatically (Dye et al., 2002; Mukherjee et al., 2004). In addition, although multidrug combinations treat TB successfully, the used multidrug regimens are ineffective against MDR-, XDR-, and TDR-TB and have not prevented transmission of disease in endemic regions due to high infectivity rate and lack of rapid diagnostic tests to detect active TB infection.

The ability of the mycobacterium to enter latency with no clinical symptoms of disease leads to poor, untimely diagnosis and treatment, which negatively impacts TB elimination strategies in the United States and many other countries (ATS/CDC/IDSA, 2000, 2005). The tuberculin test (TST), which is a conventional method for detection of *Mtb* infection, is known to have important limitations. TST was shown to cross-react with Bacille-Calmette-Guerin (BCG) vaccine and with non-TB mycobacterial (NTM) species and is particularly difficult to interpret in BCG-vaccinated foreign-born individuals from TB-endemic countries (Agger and Andersen, 2002; Shah et al., 2012). Also, inter-reader variability in result interpretation affects the outcome of TST specificity, affecting the treatment schedule and therefore the overall control of disease.

Clearly, better preventive measures that block *Mtb* transmission are needed, including vaccines that prevent infection (prophylactic, infection-preventing) or prevent further development of disease into active state (therapeutic, disease-blocking).

## Immune response to TB and its implication for vaccine development

The often-encountered difficulties surrounding the development of vaccines against TB are primarily associated with the limited knowledge of the protective immunity that is needed to clear the infection as well as the lack of identification of antigens that when targeted by the immune response will result in protection against disease development.

After inhalation of *Mtb* through aerosol particles, the mycobacteria are phagocytosed by alveolar macrophages and dendritic cells (DC) and subsequently transported to the site of infection, mainly the lungs. Inside the macrophage, *Mtb* is trapped in the phagosome where it replicates until the innate immune response activates the macrophage in an effort to control the pathogen. During this time, the bacteria are capable of arresting phagolysosome formation, through a few known mechanisms, preventing bacterial death from high pH and hydrolytic enzymes present in phagolysosomal compartment. In DC, the pathogen is carried to the draining lymph nodes, where *Mtb*–derived antigens are presented to T lymphocytes and *Mtb* antigen-specific T cells that are generated. These cells recirculate and induce formation of granulomas in the lung. The granuloma is comprised of a large number of immune cells that altogether with a fibrous cuff restrain bacterial cells from spreading. Although the innate immune response slows down the physiological dynamics of *Mtb*, it rarely leads to complete killing of the bacteria, and most often it promotes latency, whereby the bacteria is contained in the granuloma.

TB vaccine candidates should elicit cellular immune responses that are important in controlling *Mtb*. T lymphocytes are generally believed to mediate immunity against TB based both on animal models of infection and human data. Interferon-γ (IFN-γ) producing CD4+ T helper cells (Th1) and antigen-specific CD8+ T cells clearly play an important role in immunity against TB (Cooper et al., 1993; Flynn et al., 1993; Munk and Emoto, 1995; Bastian et al., 2008). The MHC class I restricted T cells also participate in resistance to *Mtb* infection (Flynn et al., 1992). This resistance appears to be mediated by grazymes A and B and by granulysin (Canaday et al., 2001). Tumor necrosis factor-α (TNF-α) and p55 receptor expression are associated with TB resistance (Flynn et al., 1995). CD4+ Th2 cells do not generally participate in mediating protection against TB, but a role for B cells in host defense is becoming apparent (Maglione and Chan, 2009) as B cell deficient mice were found to be more susceptible to TB (Vordermeier et al., 1996; Maglione et al., 2007).

IL-17-producing T helper cells (Th17 cells) have been implicated in the early phase adaptive immunity and host defense against *Mtb* (Khader et al., 2007). In addition, Th17 cells were also implicated in human and murine responses to BCG vaccination (Scriba et al., 2008; Cruz et al., 2010). *Mtb* challenge of mice vaccinated with BCG resulted in an increase in interleukin IL-17, TNF-alpha, IL-6, and MIP-2 expression along with lung tissue damage (Cruz et al., 2010). Based on the possible role of Th17 in *Mtb* infection, it is proposed that a protective TB vaccine will need to induce both Th1 and Th17 memory responses. It is hypothesized that the vaccine-elicited Th17 cells will be sequestered in the lung and upon subsequent challenge with *Mtb*, the Th17 memory cells will proliferate rapidly, producing IL-17 and triggering the local expression of chemokines that will recruit protective Th1 cells for the clearance of *Mtb* in the lungs. Therefore, a Th17 response will clearly be beneficial since it will accelerate the recruitment of Th1 cells for rapid bacterial clearance. Current vaccine studies are now focusing on IL-17-producing memory cells in the hopes of improving vaccine strategies against TB in humans.

### Innate immunity

There is a highly probable link between innate and adaptive immune response to *Mtb* and a better understanding of this interaction is critical in the development of an effective vaccine. The γ/δ T cells respond rapidly to microbial epithelial invasion and are considered a component of innate immunity (Haregewoin et al., 1989; Constant et al., 1994). γ/δ T cells produce cytokines such as IFN-γ, which is important for inducing type 1 immunity. BCG immunization induces human memory γ/δ T cells characterized by a more rapid and potent secondary responses, which suggest that these cells have a direct adaptive immune role in mycobacteria infections in humans (Spencer et al., 2008).

Another component of innate immunity to mycobacterial infections are mucosal associate invariant T cells (MAIT) (Porcelli et al., 1993). Both TB-exposed and unexposed individuals have *Mtb*-reactive MAIT cells (Gold and Lewinsohn, 2011). MAIT cells are thought to be innate T cells based on their restricted expression of the semi-invariant Va7.2 T cell receptor (TCR) and their activation via the non-polymorphic HLA-Ib molecule MR1. Because MAIT are capable of producing IFN-γ directly *ex vivo* in response to *Mtb*-infected cells, MAIT may play a role in the control of the bacterium. Questions remain, however, whether MAIT cells represent an *Mtb*-reactive innate T cell population that can supply an early source of IFN-γ in the innate control of TB disease as well as providing aid in the acquisition of an optimal adaptive Th1 immune response.

Apoptosis and autophagy are innate immune defense mechanism against mycobacteria by possibly suppressing bacterial replication and promoting DC presentation of bacterial antigens to T cells for better T cell induction and protection *in vivo*. The *Mtb* pathogen, however, has evolved to evade death by apoptosis of macrophages and neutrophils and this evasive maneuver by *Mtb* is thought to contribute to its virulence (Keane et al., 2000; Blomgran et al., 2012). Infection of macrophages with “pro-apoptotic” *Mtb* mutants (e.g., Δ*nuoG* and Δ*secA2*) leads to enhanced apoptosis and mice infected with the mutants showed augmented induction of CD4+ and CD8+ T cells (Hinchey et al., 2007; Blomgran et al., 2012).

Autophagy is an essential host defense mechanism against microbes and plays an important role in host innate and adaptive immunity. Activation of autophagic pathways in macrophages causes mycobacterial phagosomes to become mature phagolysosomes, which can compromise the survival of intracellular mycobacteria. However, *Mtb* has evolved mechanisms to suppress autophagic pathways in macrophages (Deretic et al., 2009).

Since the correlates of protective immunity against TB are not known, it is imperative to conduct human studies that will enable us to identify host and bacterial determinants that play critical roles in protection against TB infection and disease. A current prospective study led by Willem Hanekom and colleagues have established a cohort of 6363 South African adolescents. 53% were infected with *Mtb* at baseline and during 2 years of follow-up, 76 of these participants developed TB disease. Blood samples were collected and will be used to compare host responses between individuals who developed disease and those who have remained healthy. The availability of this cohort will allow us to address host responses that are involved in TB. Initial studies of this cohort showed that high bacterial load was associated with impairment of antigen-specific T cell responses in *Mtb*-infected individuals (Day et al., 2011). Knowledge of host determinants of protection against TB disease could impact TB control not only in the design of new vaccines but also prophylactic therapy for infected persons.

## Existing TB vaccine—BCG

Despite immense efforts in TB vaccine research we continue to immunize with *Mycobacterium bovis* bacillus Calmette-Guerin (BCG) vaccine (Calmette, 1931), which was approved for human use at the beginning of the 20th century. BCG was proposed as a live vaccine against TB by Albert Calmette and Camille Guérin only few years after the discovery of the intracellular pathogen *Mtb* as the causative agent of TB by Robert Koch in 1890 (Calmette et al., 1907). *M. bovis* BCG was generated after continual passaging of the parental *M. bovis* strain for 13 years (a total of 230 passages) in media containing bile, which resulted in an attenuated strain with reduced virulence in animals (Calmette and Guerin, 1920). BCG vaccine production started in 1927 that then led to generations of daughter BCG strains with different genomic composition (Behr, 2002). BCG vaccine is administered worldwide as a single intradermal inoculation dose.

The use of heat-inactivated or live attenuated pathogens was the preferred choice for many years in traditional vaccinology since they contain a vast array of antigens. Because of this, a broad and diverse immune response is induced resulting in protection against the pathogen. However, BCG vaccination affords only partial protection in humans. BCG protects against miliary TB and meningitis TB in infants (Rodrigues et al., 1993; Trunz et al., 2006), but fails to protect against pulmonary and latent TB in adults of both sexes and all ages, including children (ICMR, 1999; Narayanan, 2006). The variable efficacy of BCG, ranging from 0 to 80% in randomized control trials is attributed to a number of factors, including geographical location of human population, loss of virulence genes essential in protective immunity, an insufficient induction of CD8+ T cell response, exposure to environmental (non-tuberculous) mycobacteria or helminthic infection prior to BCG vaccination (Fine, 1988; Agger and Andersen, 2002; Lalor et al., 2009; Rowland and McShane, 2011).

## TB vaccines in clinical trials

Due to protection conferred by BCG in childhood, current vaccines strategies in clinical trials are either focused on engineering BCG to be more immunogenic or boosting prior BCG vaccination with new vaccine regimens in the hopes of increasing protection (Table 1). There are also a few new live vaccines being tested to entirely replace BCG due to the associated risk of infection with the viable vaccine in TB-HIV population (von Reyn et al., 2012).

**Table 1:**
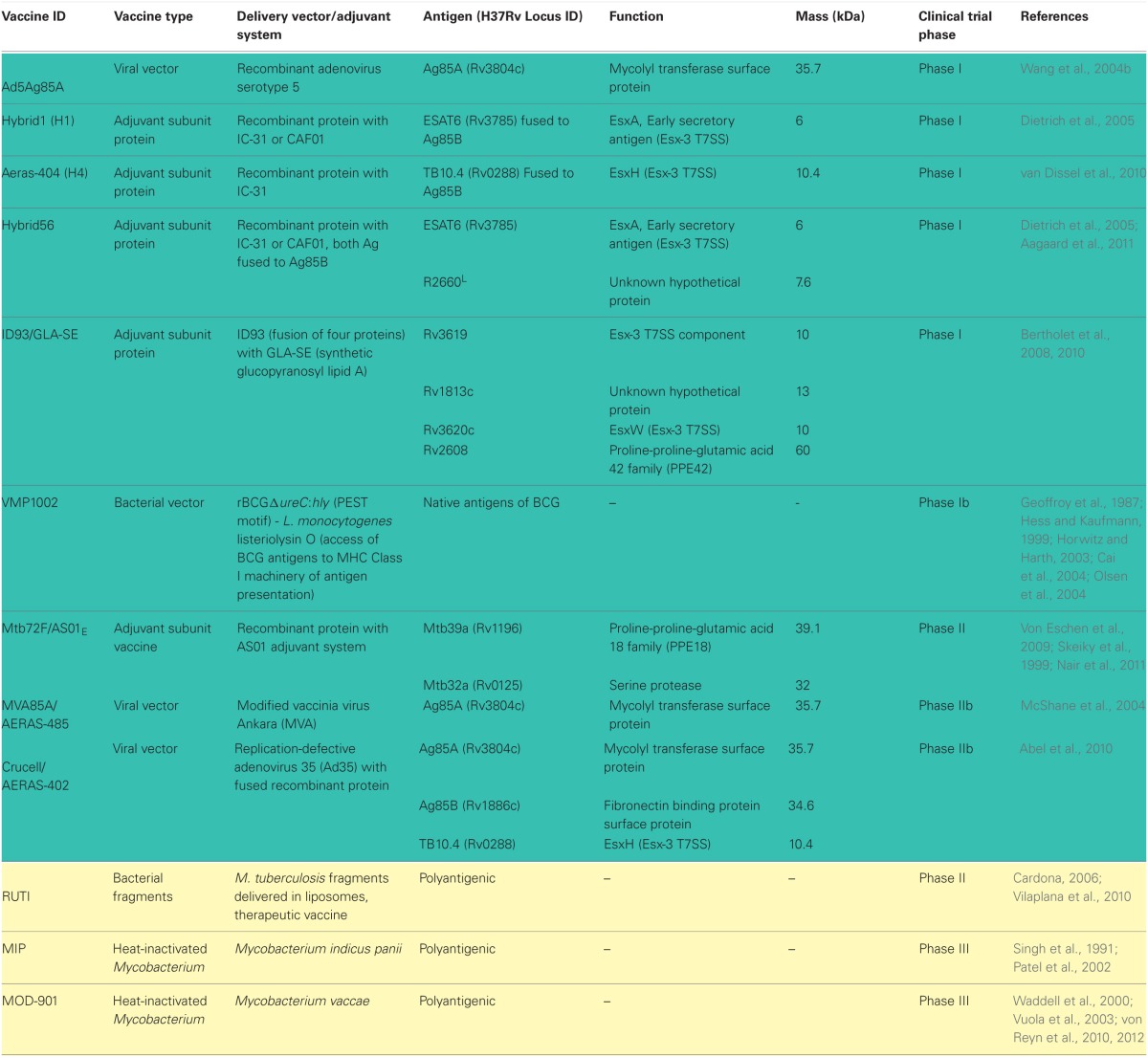
**TB vaccine candidates in clinical trials and their antigens**.

Cells in green are prophylactic vaccines and cells in yellow are therapeutic vaccines.

### Live mycobacterium-based vaccines aiming to replace BCG

New-generation mycobacterial vaccine vectors utilize current BCG as backbone to express known T-cell immunogens from virulent *Mtb* or mutants of BCG that are capable of escaping the phagosome to induce CD8+ T cell response are being tested as vaccine candidates. BCG shares many proteins in common with *Mtb*, however many of these proteins are not well recognized by vaccinated humans or animals. One such antigen is the molecule known as antigen 85 B (Ag85B), a major secretory protein produced by *Mtb* previously shown to induce protection to TB in mice and guinea pigs (Horwitz et al., 1995). Although BCG produces this protein, little or undetectable immune response to this molecule is observed after vaccination. However, a recombinant BCG vaccine (rBCG30) overexpressing and secreting ~5.5 fold more Ag85B than conventional BCG induced an order of magnitude greater immune response when compared to the parental BCG vaccine. A Phase I clinical trial with rBCG30 has been successfully completed (Horwitz et al., 2000; Hoft et al., 2008). Another interesting recombinant BCG-based vaccine is VMP1002 (Kaufmann, 2012; Kaufmann and Gengenbacher, 2012; Ottenhoff and Kaufmann, 2012). VMP1002 was engineered using specific properties of listeriolysin O, a secreted thiol-activated cholesterol-binding hemolysin from *Listeria monocytogenes* (Table 1) (Geoffroy et al., 1987). This protein forms multimeric forms that allow engineered VMP1002 cells to escape from phagolysosomes (pH5.5) to the cytosol of the host, where the microbial vaccine antigens can be processed by the MHC Class I machinery and then presented to CD8+ T cells (Geoffroy et al., 1987; Hess and Kaufmann, 1999; Horwitz and Harth, 2003; Cai et al., 2004; Olsen et al., 2004).

### Booster vaccines to augment BCG immunity

The rationale behind this strategy is to boost anti-TB immunity induced by prior vaccination with BCG. The goal is to continue implementing BCG immunization in neonates but subsequently boost at an older age with another TB vaccine regimen to augment immunity and protection afforded by prior BCG vaccination. Several booster vaccines could be used including recombinant viral poxviruses and adenoviruses vectors, which are highly immunogenic and currently being evaluated as vaccine delivery systems for HIV/AIDS and other diseases (Cosma et al., 2007). MVA85A is a replication-deficient vaccinia virus Ankara (MVA) used as delivery system for the mycobacterial antigen 85A, which augmented BCG-induced immunity in humans (McShane et al., 2004).

A replication-deficient strain of adenovirus used a delivery vector elicits high magnitude and functional CD4+ and CD8+ T cell responses to a vaccine candidate (McShane et al., 2002). Indeed the engineered Ad5Ag85A, a recombinant adenovirus serotype 5 (Ad5) vaccine vector expressing the *Mtb* antigen 85A is currently being tested in a phase I safety and immunogenicity study in BCG-vaccinated and-nonvaccinated healthy adults in Canada (Wang et al., 2004b). Another variation of this vaccine uses a non-replicating adenovirus serotype 35 expressing antigens 85A, 85B, and TB10.4 and is in clinical trial under the name of AERAS-402 (Abel et al., 2010). The advantage of Ad35 over other delivery viral vectors is the low frequency of anti-adenovirus neutralizing antibodies and low-levels of pre-existing immunity found in humans.

Another strategy to boost BCG-induced immunity is to use protein-based subunit vaccines as boosting agents. Subunit vaccines currently in clinical trials include Mtb72F/ASO1/ASO2A, which consists of a 72 kDa recombinant protein containing Mtb32 (Rv1196) and Mtb39 (Rv0125) antigens previously shown to induce strong CD4+ and CD8+ T cells responses in laboratory animals but more importantly, in healthy, PPD-positive individuals (Skeiky et al., 1999, 2000, 2005). The recombinant polyprotein is used with the AS02A adjuvant, an oil-in-water emulsion with 3-deacylated monophosphoryl lipid A (MPL) and QS-21 detergent (Pichichero, 2008). Another vaccine candidate in clinical trial is AERAS-44, which is a fusion protein called Hybrid 1 consisting of antigens 85B (Olsen et al., 2004) and TB10.4 (Rv0288) (Dietrich et al., 2005). The H1 vaccine combined with adjuvants IC31, a synergistic combination of single-stranded oligodeoxynucleotide and a peptide (KLKL5KLK) that induces the Toll-like receptor-9 (TLR9) (Schellack et al., 2006) had promising preclinical data in that the vaccine elicited Th1 responses and increased protection against *Mtb* in mice (van Dissel et al., 2010; Aagaard et al., 2011). H56-IC3 is another candidate vaccine in early human trials. This promising vaccine formulation contains novel latency-associated TB antigen, Rv2660c, along with Ag85B, ESAT-6, and the IC31 adjuvant (Aagaard et al., 2011).

### Therapeutic vaccines

Therapeutic vaccines are being developed to treat people already infected with *Mtb* as adjunct therapy. However, immunotherapy with *Mtb* bacterial components poses a health risk since TB patients when inoculated with high doses of *Mtb* antigens can have adverse reactions similar to a tuberculin shock, which was first described by Robert Koch over 100 years ago (Rook and Stanford, 1996). More recently one of us has shown that a single recombinant molecule of *Mtb* can trigger toxemic reaction in guinea pigs previously infected with *Mtb* (Reece et al., 2005). Therefore, careful selection of the candidate antigen to be used in immunotherapeutic protocols is highly recommended.

Nonetheless, immunotherapeutic vaccines are being tested in clinical trials. A candidate therapeutic vaccine that is under evaluation is RUTI, a heat-inactivated *Mtb* cellular fragment, designed to shorten the chemotherapy of LTBI (Cardona, 2006; Vilaplana et al., 2010, 2011). This phase II study compares three different doses of RUTI given after 1 month of isoniazid in HIV-positive and HIV-negative adults showed that the vaccine was well tolerated, with the most common adverse events being mild injection-site reactions. Efficacy of RUTI vaccine regimen is currently being evaluated.

Another therapeutic vaccine candidate is ID93/GLA-SE (Bertholet et al., 2008, 2010). This product is a fusion of four mycobacterial antigens, including one latency antigen into a synthetic TLR-4-agonist, glucopyranosyl lipid A (GLA) adjuvant that combines an innate signal with a potent Th1-inducer (Table 1) (Coler et al., 2011; Pantel et al., 2012). Testing in hyper-susceptible inbred mouse strain (SWR/J) and cynomolgus monkey models showed that ID93/GLA-SE vaccination augmented the therapeutic effect of rifampin and isoniazid (Coler et al., 2012). Therapeutic vaccination with ID93/GLA-SE reduced lung bacillary loads and pathology and shortened the treatment duration by one-third when compared to the treatments with antimicrobials alone, although the magnitude of therapeutic vaccination in monkeys appeared to be modest.

Finally two heat-inactivated mycobacterial strains *M. indicus pranii* (MIP) (Singh et al., 1991; Patel et al., 2002) and *M. vaccae* (Waddell et al., 2000; Vuola et al., 2003; Xu et al., 2009; Yang et al., 2011) have been tested in combination with the chemotherapy for *Mtb* infection. Administration of various doses of MIP subsequent to TB chemotherapy seems to decrease the inflammatory responses and lead to amelioration of lung pathology in tested animal models (Gupta et al., 2012). *M. vaccae* administered in a multiple-dose series format promoted significant protection against TB in HIV-infected adults who have been vaccinated with BCG during childhood (von Reyn et al., 2010, 2012). Therefore this protocol represents a potential immunotherapeutic strategy against TB for a large population of patients that are co-infected with TB and HIV.

## Novel TB vaccine approaches

Despite the testing of several new TB vaccine candidates in human trials, there is no guarantee that any of these vaccines will be better than BCG. There are still gaps in our knowledge of the human correlates of protective immunity and our understanding of host-pathogen relationships especially immune evasion strategies employed by *Mtb*. The more insights we gain in these areas of TB research the better we are equipped at creating an effective vaccine against TB.

**Figure 1:**
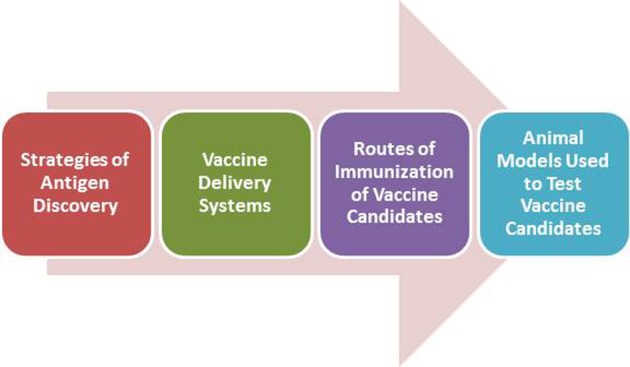
**Approaches of vaccine development to TB discussed in the present review**.

In the next sections we will emphasize novel approaches and strategies in TB vaccine development, highlighting scientific issues that need to be addressed in this complicated field of vaccine development (Figure 1).

### New antigen discovery strategies

**Figure 2:**
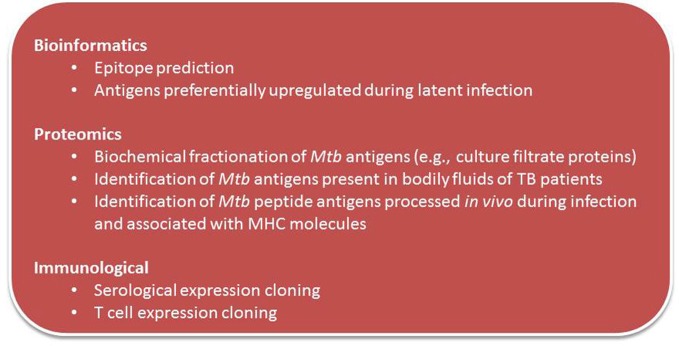
**Strategies of antigen discovery of TB vaccine candidates**.

Traditional methods for identification of *Mtb* vaccine candidate molecules including those that identified candidate antigens that found in culture filtrate (CF) were later confirmed to elicit immune responses in animal hosts infected with TB. Figure 2 summarizes current and novel antigen discovery strategies of candidate vaccine for TB.

New technologies and developments in bioinformatics have made possible the search for novel vaccine candidate antigens *in silico*, based on genomic sequence of the pathogen (Rappuoli, 2000; Rappuoli et al., 2011). This “reverse vaccinology” is an unbiased approach that uses known homologous sequence data to search for multiple immunogenic antigens. Seventy new *Mtb* class I CD8+ T cell epitopes were identified and the immunogenicity of 18 of them have been assessed with tetramers. There was broad IFN-γ, IL-2, and TNF-α responses to these epitopes on cured TB patients suggesting that there is a much broader CD8+ response than previously appreciated (Tang et al., 2011).

Based on findings that human T cells from TB patients were shown to recognize hyperconserved epitopes that are thought to benefit the *Mtb* pathogen (Ernst, 2012), one can hypothesize that human T cell recognition of previously discovered hyperconserved epitopes are non-protective and that those that are variable are more closely associated with protective immunity. On this premise, Joel Ernst and colleagues seek to identify immunogenic variable T cell epitopes using *in silico* analyses that may be protective from sequences of 180 phylogenetically-diverse strains of *Mtb*, and the most diverse regions of the *Mtb* genome will be identified. T cell responses will be assayed to synthetic peptides having sequences matching those in the subject's infecting isolate, to identify which of the predicted epitopes are true targets of human T cell recognition. It is anticipated a large set of immunogenic variable epitopes will be discovered and potentially could be used as vaccine immunogens to elicit protective T cell responses.

#### Identifying vaccine antigens from active TB

Antigens that are recognized by host immune cells during active TB, when *Mtb* is replicating, are potential immunologic targets for controlling the pathogen. Strategies to identify these important antigens represent an alternative to current approaches to vaccine antigen discovery. The focus of our laboratory for a number of years has been to search for antigens recognized by host immune cells during active *Mtb* infection. We have accumulated a portfolio of potential vaccine antigens present in various bodily fluids of humans and animals infected with TB (Mukherjee et al., 2005; Kashino et al., 2008a; Napolitano et al., 2008). Antigens expressed and secreted in urine of patients with pulmonary TB (Kashino et al., 2008b) and infected mice (Mukherjee et al., 2005) were identified using 2D-gels followed by mass spectrometry. Four unique peptide-homologs to *Mtb* proteins were identified in the urine of pulmonary TB patients (Kashino et al., 2008b; Napolitano et al., 2008) with one of the immunogens a molybdopterin biosynthesis protein (MoeX, Rv1681; MT_1721) unique to *Mtb* complex, and the other three antigens are present in all mycobacterial members with ~70% homology. These are currently being evaluated and preliminary preclinical studies showed that the subunit and DNA vaccine expressing MT1721 was highly immunogenic, capable of eliciting both CD4+ Th1 and CD8+ T cell responses (Cayabyab et al., 2012).

#### Protective antigens that will prevent latent TB

To develop an efficacious TB vaccine, vaccinologists must find a solution to the problem of latency and immune evasion in vaccine design. One logical approach is to identify antigens preferentially expressed during latency. Several promising vaccine antigens have been identified that could be targets of the host immunity during chronic infection. A subunit vaccine (H56) containing the combination of early secretory Ag85B/ESAT-6 and latent (Rv2660c) antigens protected mice against latent and reactivation TB and the H56 vaccine lowered bacterial burden in mice already infected with *Mtb* (Aagaard et al., 2011).

#### Identifying antigens that induce CD8+ T cells

Numerous studies suggest that CD8+ T cells will be needed to protect against TB. *Mtb* efficiently induces Ag-specific CD8+ T cells to various MHC I-restricted epitopes (Billeskov et al., 2007; Begum et al., 2009; Woodworth et al., 2011), and *Mtb*-specific CD8+ T cells are important in protection to mice in secondary *Mtb* infection (Wang et al., 2004a; Wu et al., 2008). A novel strategy to identify CD8+ T cell antigens is a major research effort of our and other laboratories. To discover potential CD8+ T cell vaccine antigens, we eluted *Mtb*-specific peptides bound to major histocompatibility complex (MHC) class I molecules from spleens of infected mice and identified several candidate antigens. Preliminary immunogenicity studies revealed that one of the identified T cell antigens, MT0401, was highly immunogenic and recognized in *Mtb*-infected mice. Vaccination of mice with the new recombinant VRC8400 DNA vaccine vector and Ad5 vectors expressing the MT0401 antigen elicited potent CD4+ and CD8+ T cell responses (our unpublished observations). The protective efficacy of the vaccine vectors expressing MT0401 antigen in mice and guinea pigs are currently being evaluated.

### Novel adjuvants to stimulate vaccine-induced TB immunity

**Figure 3:**
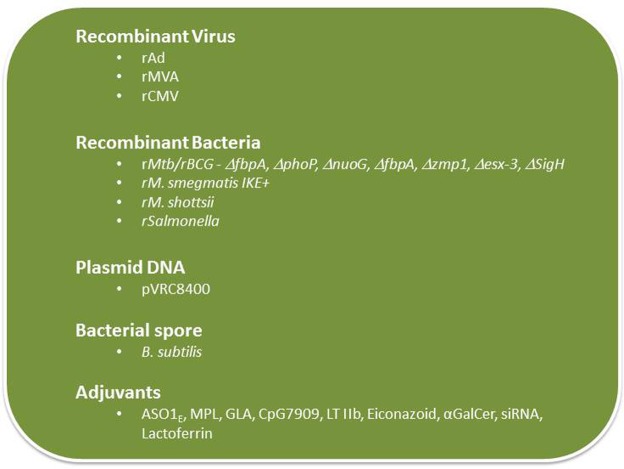
**Delivery systems used in vaccine development for TB**.

There are only a few adjuvants approved for human use and subunit vaccines mixed with those adjuvants largely elicit CD4+ T cell responses and humoral immunity and stimulate poor CD8+ T cell responses. Since there is general consensus that an effective vaccine against *Mtb* will require both CD4+ and CD8+ T cells, and perhaps to some extent the humoral arm of the immune response, it is critical that new adjuvants be tested that will be more potent than formulations currently used clinically (Figure 3).

Glucopyranosyl-lipid A (GLA) is a non-toxic, synthetic molecule, homogeneous to monophosphoryl lipid A from *Salmonella minnesota* (MPL) that has been recently developed by the Infectious Disease Research Institute (IDRI), Seattle, WA. GLA formulated with squalene forms a stable oil-in-water emulsion (GLA-SE). This formulation, similar to MPL-SE, is a Th1-stimulatory adjuvant shown to be effective in preventing and reducing disease caused by Leishmania parasites (Bertholet et al., 2009). A GLA-SE formulation includes a polyprotein TB vaccine candidate, which includes four *Mtb* antigens (Rv2608, Rv3619, Rv3620, and Rv1813) was recently developed and tested by scientists at IDRI. This formulation showed promising results in protection experiments performed in mice challenged with virulent (Windish et al., 2011). IDRI has announced a new partnership with Aeras to develop this candidate and a phase I trial is scheduled to start this year (http://www.idri.org/press-5-9-12.html).

Carbomers (polymers of acrylic acid) have been used in the pharmaceutical industry to achieve controlled release of medications in tablets and as a bioadhesive in mucosal applications. Immunization with a soluble antigen containing a carbomer-based adjuvant induced antigen-specific T cell producing both Th1 and Th2 cytokines including high titers of IFN-γ, IL-2, and IL-4, and drove a Th1 isotype-switched antibody response (Krashias et al., 2010; Dey et al., 2012). The underlying mechanisms responsible for the adjuvant properties of carbomers and the functionality and the protective capacity of the T cell responses induced by subunit antigens mixed in carbomers need to be investigated prior to the use of carbomers as a TB vaccine adjuvant.

TLR-9 control multiple DC functions and adaptive immune responses in addition to their role in innate immunity. TLR ligation represents a novel approach to augmenting TB vaccines. The CpG 7909, a TLR9 ligand, has been shown to be a potent adjuvant to pneumococcal vaccines in early human immunogenicity studies (Sogaard et al., 2010) and is now being tested in Phase I trials as a vaccine adjuvant in the treatment of cancer (Adams, 2009). Subunit vaccines with CpG 7909 is expected to generate a strong broad adaptive immune response, including T helper 1 and CD8+ T cells, important feature of a vaccine needed to protect against TB.

Elicitation of protective mucosal immune responses to mucosal pathogens such as *Mtb* is hindered by endogenous regulatory systems that suppress immune responses to foreign antigens on mucosal surfaces. To bypass those regulatory systems, mucosal adjuvants are needed and a strong mucosal adjuvant, LT-IIb (T13I), a non-toxic type II heat-labile enterotoxin currently being tested (Lee et al., 2011; Casey et al., 2012) could potentially be used as an adjuvant to enhance subunit TB vaccines.

Mycobacterium-induced apoptosis is an innate defense mechanism that can prevent the bacteria from establishing in the host, perhaps because apoptosis can promote DC-mediated presentation of bacterial antigens to T cells and generation of adaptive anti-bacterial immunity. How apoptosis is regulated and restricts *Mtb* replication and whether it can be manipulated to enhance vaccination is the focus of current investigations (Behar et al., 2011a,b). Sam Behar and colleagues are exploring eicosanoids as a pro-apoptotic agent since these compounds play a role in immunity to mycobacteria by regulating activation of infected macrophages leading to control of intracellular bacterial replication. An attractive vaccination strategy would be to pharmacologically manipulate the eicosanoid biosynthetic and cell signaling pathways to enhance apoptosis of infected macrophages. By promoting apoptotic death, it may be possible to increase the safety and the efficacy of attenuated bacterial vaccines.

The glycosphingolipid α-galactosylceramide (α-GalCer, also known as KRN7000) is a synthetic analog of the marine natural product agelasphin that activates both human and mouse invariant natural killer T cells to produce immune-regulatory cytokines (Sogaard et al., 2010). BCG with stably incorporated α-GalCer was found to stimulate increased maturation of DCs and augmented the priming of CD8+ T cell responses that correlated with better protection against *Mtb* challenge in mice (Venkataswamy et al., 2009). The clinical utility of α-GalCer as a TB vaccine adjuvant needs to be determined.

Vaccine adjuvants are needed to activate DC for T cell priming, but these adjuvants simultaneously induce the expression of suppressive genes including T regulatory cells and inhibitory receptors (e.g., PDL1 and PDL2) and their signaling pathway molecules (e.g., SOCS1, SOCS2, and IRAK-M). To maximize the immunostimulatory potential of vaccines, it may be necessary to downregulate inhibitory receptor signaling pathways activated by adjuvants and vaccine vectors. The use of siRNA that will block the immunosuppressive activity of inhibitory receptors as a component of a vaccine formulation is currently being tested (Lee, 2012a). A new method for *in vivo* gene silencing being explored makes use of Glucan encapsulated siRNA Particles (GeRP), which is a packaging system of charged molecules, including protein antigens and adjuvant compounds along with siRNA inside the glucan shells (Aouadi et al., 2009). The resulting particles allow co-delivery of a complete vaccine formula to a DC via the receptor Dectin-1. The silencing capability of GeRP and its ability to augment vaccine-induced immune responses are being assessed.

Lactoferrin, an iron binding glycoprotein, has been previously shown to promote maturation of T- and B-lymphocyte and immature DC, and enhance the ability of macrophages and DCs to stimulate antigen-specific T-cells. Lactoferrin is currently being tested as an adjuvant because of its ability to enhance the generation of antigen-specific DTH responses (Actor et al., 2009). Lactoferrin mixed with BCG vaccine promoted host protective responses that surpasses activity of the BCG vaccine alone as determined by decreasing pulmonary pathology upon challenge with virulent *Mtb* (Hwang et al., 2011). Production of IL-17 and IFN-γ is increased while IL-10 production is decreased in mice vaccinated with BCG/lactoferrin compared to the group vaccinated with only BCG. This study illustrates adjuvant activity of lactoferrin by enhancing BCG immunogenicity.

#### Novel mycobacterial vectors

The next generation of mycobacterial vectors should be more immunogenic than BCG by eliciting better innate and adaptive immune responses that prevents primary TB as well as LTBI through effective elimination of the *Mtb* bacilli. The use of gain-of-function or loss-of-function screens has enabled the identification of several classes of potentially more immunogenic BCG mutants and mycobacterial vectors. One class of mutants are those that inhibit apoptosis of macrophages and Th1 response which has been linked to virulence and immune evasion by *Mtb*. Using gain-of-function screens, several anti-apoptotic genes have been identified including *nuoG* (Velmurugan et al., 2007). Deleting anti-apoptotic genes should result in the creation of the so-called “pro-apoptotic” attenuated mycobacterial vaccine vectors that should be more immunogenic than BCG.

*Mtb* and to some extent BCG has the ability to actively suppress immune responses including the induction of Th1 cytokines. Therefore, creating mutants of BCG and other mycobacterial vaccine vectors that lack this activity could be excellent TB vaccine candidates. Several of these mutants have been described. The William Jacobs, Jr. laboratory (Albert Einstein College of Medicine) has recently identified gain-of-function mutants that can induce IL-2 and IL-12 cytokines that promote Th1 and protect mice from *Mtb* challenge (Derrick et al., 2012). A deletion mutant of *Mtb* in the 85A gene (Δ*fbpA*) resulted in attenuation of the pathogen and appears to be highly immunogenic (Saikolappan et al., 2012). Unlike wild type H37Rv and to a certain extent BCG, Δ*fbpA* is not suppressive for macrophages or DC and enhances antigen presentation and the priming of Th1 cells more effectively than BCG in mice leading to a better protection. Mutants of the *phoP* response regulator in *Mtb* were found to be more immunogenic than wild-type *Mtb*. When used as a vaccine, the mutant was found to protect better than BCG and the protection was associated with increased frequency and persistence of antigen-specific central memory CD4 T cells (Nambiar et al., 2012).

Deletion of microbial antioxidants could lead to the development of more immunogenic BCG vaccine vectors. Indeed, elimination of duplicated alleles encoding the oxidative stress sigma factor H (SigH) in BCG and reducing the activity and secretion of iron co-factored superoxide dismutase resulted in better cellular responses to BCG. Compared to mycobacteria-specific immune responses in mice after vaccination with BCG, the modified vaccine (BCGΔ*secA2*Δ*sigH*) induced greater IL-12p40, RANTES, and IL-21 expression in the spleens of mice post-immunization, more cytokine-producing CD8+ lymphocytes at the peak of the primary immune response, and more IL-2-producing CD4+ lymphocytes during the memory phase. Moreover, BCGΔ*secA2*Δ*sigH* induced stronger secondary CD4+ lymphocyte responses and greater clearance of challenge bacilli than the conventional BCG (Sadagopal et al., 2009).

Another rBCG strategy was to delete from the mycobacterial genome the gene involved in phagosome maturation, *zmp1* gene, (Johansen et al., 2011). The rBCGΔ*zmp1* was found to be more immunogenic than wild-type BCG in the murine model, suggesting that promoting phagosome maturation and lysosomal delivery of BCG enhances immunogenicity.

An alternative strategy to create more immunogenic mycobacterial vector is to generate gain-of-function mycobacterial mutants capable of inducing autophagy (Lee, 2012b). Autophagy, an important host defense pathway, has an essential role in both innate and adaptive immunity. However, many microbes including *Mtb* have evolved mechanisms to suppress autophagic pathways in macrophages that cause phagosomes to mature into phagolysosomes that is detrimental to the pathogen. Generation of “pro-autophagic” mutants of mycobacteria may have significant application in the development of effective, safe and persistent TB vaccines.

In addition to genetically modified BCG mutants, other mycobacterial species are being considered as vaccine vectors against TB and other diseases such as HIV because certain species were found to be more immunogenic than BCG and may have a better safety profile. The commensal non-pathogenic *M. smegmatis* is being developed as a vaccine delivery system because it is fast and easy to grow in culture, genetically tractable, can express large amounts of foreign antigen and capable of inducing robust T cell and antibody responses either as a standalone or in heterologous-prime boost strategies (Cayabyab et al., 2006; Yu et al., 2006; Hovav et al., 2007; Sweeney et al., 2011). *M. smegmatis* with an intact *esx-3* locus is lethal to mice, while a deletion mutant Δ*esx-3* (IKE strain) was controlled and cleared by MyD88-dependent immune response (Sweeney et al., 2011). Introduction of *Mtb esx-3* genes back in *M. smegmatis* (IKEPLUS) resulted in protection against challenge with virulent *Mtb*, better than BCG. Attempts at testing IKEPLUS in early human trials are underway.

TBVac85, an experimental live, attenuated intranasal TB vaccine is cold-adapted, temperature-restricted engineered *Mycobacterium shottsii* (Quinn, 2012). This mutant expresses Ag85B and contains a highly immunogenic cell wall and antigenic similarity to *Mtb*. TBVac85 is likely to be safe in immunocompromised hosts due to temperature-restricted growth attenuation. Preliminary studies in laboratory animals showed that TBVac85 is safe and immunogenic.

#### Other bacterial vaccine vectors

The importance of long-term and mucosal antigen delivery was assessed by the use of *Mtb* antigens delivered by live recombinant Salmonella vaccine consisting of plasmids expressing fusion proteins of type 3 secretion systems from Salmonella and *Mtb* antigens ESAT-6 and CFP-10 (Juarez-Rodriguez et al., 2012). In this model, mice receiving an oral vaccine exhibited delayed lysis and a regulated delayed antigen synthesis, resulting in protection against TB similar to protection induced by BCG.

Another novel delivery vector is *Bacillus subtilis* spores. *B. subtilis* spores can withstand extreme conditions of low pH, desiccation, UV irradiation and high temperatures and are harmless to humans, making them an ideal needle-free delivery system of immunogens (Amuguni and Tzipori, 2012). *B. subtilis* spore expressing Ag85B and ESAT-6 is currently under development as a TB vaccine in preclinical stages (Dhandayuthapani, 2012).

### Heterologous prime-boost strategies

Recombinant BCG and other mycobacteria (i.e., *M. smegmatis*) or more-immunogenic BCG mutants are being combined with other vaccine vector prototypes in a heterologous prime-boost strategy to induce more robust immune responses than homologous prime-boost vaccinations. Other vaccine vectors are also being used to boost *Mtb*-specific immune responses, elicited from previous routine BCG vaccinations. In addition to the aforementioned viral vectors, Thomas Evans and Aeras researchers are developing recombinant adenoviruses serotypes 4, 26, and the chimeric rAd5HVR8 as well as recombinant human CMV vectors for the delivery of TB antigens since these were found to be potent inducers of cellular immunity, including CD8+ T cell responses (Roberts et al., 2006; Alexander et al., 2012; Barouch et al., 2012). We propose a heterologous prime/boost strategy using of the DNA VRC8400 vaccine vector and the adjuvant triggering Toll-like receptor 4 agonist, GLA-SE, in a heterologous DNA prime, protein boost strategy. We found that priming mice with VRC8400 expressing the previously identified candidate vaccine antigen, rMT1721 followed by boosting with the rMT1721 subunit antigen mixed with GLA induced robust CD4+ Th1 and CD8+ T cell and antibody responses (Cayabyab et al., 2012). This protocol is currently being tested in protection experiments.

### Route of immunization

The dose and route of immunization of the candidate TB vaccine will be critical to the elicitation of protective lung immune responses against TB. Although, pre-clinical studies in vaccine development to TB have used and evaluated a variety of routes of antigen delivery (intradermal, subcutaneous, intramuscular, intra nasally, and orally) the vaccine candidates in current clinical trials have been restricted to using the parenteral routes only. Parenteral immunization has been historical successful for a variety of human vaccines to several different diseases. However, because *Mtb* is a mucosal pathogen it is imperative that a vaccine delivery system induces protective mucosal immunity in the lungs where *Mtb* initiates its infection in the alveolar macrophage. It is possible that the lack of trials using a mucosal vaccine delivery system for TB may be due to our limited knowledge on how to efficiently formulate the vaccine and deliver it to a mucosal site. In addition, it is highly debated how the route of a mucosal vaccine antigen delivery influences the homing specificity and anatomical distribution of resultant T lymphocyte responses. Since mucosal immunity and the trafficking of T cells generated not only depends on the dose and route but also heavily on the type of vaccine delivery system (Kaufman and Barouch, 2009), it is difficult to predict the quality and trafficking of mucosal B and T cells induced by a particular TB vaccine candidate. There is evidence to suggest that vaccine-elicited mucosal T cells may initially remain localized at the site of inoculation but may subsequently become broadly distributed to other mucosal compartments and/or systemically (Offit et al., 1991; Masopust et al., 2001, 2004). In humans, mucosal immunization with cholera toxin B subunit, which has been used as a mucosal adjuvant, clearly shows that the strongest response occurs at the mucosal site where the antigen/adjuvant formulation was delivered. However, potent responses are also detected in the contiguous or interconnected mucosal sites (e.g., nasal-pulmonary tract and gut-mammary gland link in lactating women respectively). Interestingly, nasal immunization also stimulates strong immune response at unrelated anatomical sites like the genital-vaginal mucosa (Holmgren and Czerkinsky, 2005). Therefore, much effort needs to be placed on the pre-clinical evaluation of mucosal immunization in vaccine development to TB, particularly using formulation that exploit the nasal route.

## Animal models to test TB vaccines

Several animal models have been developed for studies of TB disease and protection induced by candidate vaccines (Table 2). These include *Mtb* infection of mice, rats, guinea pigs, rabbits, and monkeys. The mouse TB model is preferred because of availability of immunological reagents, inbred and genetically engineered strains and low cost (Orme, 2005; Ordway and Orme, 2011). However, mice fail to display the large spectrum of pulmonary pathology seen in human infections and they do not form necrotic granulomas. Infection of guinea pigs with *Mtb*, on the other hand, closely resembles that of human infection by formation of caseating granulomas (Turner et al., 2003; Orme, 2005). Because of differences in disease susceptibility, mice are generally utilized to study anti-TB immune responses, while guinea pigs are used as a model to study progressive pathology of TB. Infected rabbits develop lung cavitary TB that resembles many aspects of human disease, including similarities in lung pathology and development of caseation (Dannenberg, 2001; Subbian et al., 2011). In addition, inoculation of rabbits with *Mtb* or BCG into the subarachnoid cistern leads to the development of a disease that clinically and histologically resembles human TB meningitis (Behar et al., 1963; Tsenova et al., 1998, 2005). The non-human primate model exhibits a large range of granulomas, from caseous, to cavitary, closely resembling the human infection (Dutta et al., 2010). Curiously and non-understandably, BCG vaccination protects cynomolgus monkeys (*M. fascicularis*) but not rhesus monkeys (*M. mulatta*) against pulmonary *Mtb* challenge (Langermans et al., 2001). However, the TB-susceptible monkey model allows for the screening of vaccine candidates that could potentially be better than BCG (Agger and Andersen, 2002).

**Table 2:** **Animal models used in vaccine development to TB**.

**Disease manifestation**	**Animal model**			
	**Mice**	**Guinea pigs**	**Rabbit**	**Non-human primates**
Primary TB	+	+	−	+
TB meningitis	−	−	+	−
Latent TB				
Cornell model	+	+	−	−
*Mtb* strain 18B model	+	+	−	−
Pulmonary TB (Adult or chronic TB)	−	−	+	+

An enormous challenge to studying latent TB infection and evaluating vaccine candidates against this form of infection is the unavailability of an adequate animal model that can mimic the human LTBI infection. The latency model generally referred to as the Cornell model was first described in the late 1950s (McCune et al., 1956, 1966a,b; McCune and Tompsett, 1956). In this model mice are inoculated intravenously (i.v.) with ~2× 10^6^ viable bacilli of virulent *Mtb* and the resultant infection is treated for 12 weeks with the anti-mycobacterial drugs isoniazid (INH) and pyrazinamide (PZA) beginning within 20 min after infection. After the 12-week antibiotic treatment, no tubercle bacilli can be cultured from the animals' organs for many months. However, at this time point, administration of cortisone (at immunosuppressive doses) at 2–3 months after the interruption of the antibiotic therapy reverts this condition, and *Mtb* can be cultured from lungs and spleens of ~50% of the animals. Despite having the advantage of achieving and maintaining for many weeks very low numbers of the tubercle bacilli within the tissues of infected mice, this model has three major limitations: (1) Dormancy is difficult to standardize because the optimal antibiotic concentration and duration of treatment to achieve low numbers of bacilli varies from experiment to experiment; (2) Only 50% of the animals successfully treated with antibiotic develop dormant infection, which imposes a major complication in the interpretation of the experiments (Lenaerts et al., 2004); and (3) Most variants of the Cornell model use high doses of immunosuppressive agents to achieve reactivation, which by definition constitutes a complication for studies designed to evaluate the host immune response during reactivation of the disease. An interesting alternative of animal model of latent TB in both mice and guinea pigs has been recently proposed (Kashino et al., 2006, 2008a). A streptomycin-dependent (auxotroph) *Mtb* strain 18b that was isolated in 1955 in Japan from a patient with streptomycin-resistant TB (Hashimoto, 1955) was utilized in these studies. The *Mtb* 18b strain, which was demonstrated to be entirely streptomycin-dependent, was unable to grow unless streptomycin is present in the growth media (Hashimoto, 1955; Honore et al., 1995). In this model of TB latency, the *Mtb* 18b strain was shown to replicate in the lungs and spleens of mice and guinea pig treated with streptomycin and upon antibiotic treatment withdrawal the bacteria stopped replicating concomitant with the over-expression of α-crystallin (Kashino et al., 2006, 2008a) which is a protein member of dormancy regulon (DosRS) and one of the most abundantly produced protein during exposure to hypoxia and nutrient starvation (Cunningham and Spreadbury, 1998; Sherman et al., 2001), leading to the conclusion that *Mtb* 18b mouse infection model mimics latent TB. The *Mtb* 18b infection was characterized by formation of granuloma and the persistence of low numbers of viable non-replicating bacilli in mice and guinea pigs for at least 6 months. It was also shown to induce resistance to reinfection with virulent *Mtb* and potent T-cell responses to native and purified recombinant *Mtb* proteins (Kashino et al., 2006). Intriguingly, after starvation for longer periods of time, the bacilli resume replication once streptomycin is added back to the culture, confirming that streptomycin is required for exiting the dormant state in this particular strain and was used recently for screening of drugs against non-replicating bacteria (Sala et al., 2010).

Finally, the recent development of humanized mice will greatly help to explore HIV/*Mtb* co-infection due to the human host tropism of HIV. A commonly used humanized mouse model is the NOD-SCID/3c null mice engrafted with human fetal liver and thymic tissue, and injected intravenously with CD34+ fetal liver cells from the same tissue source (Shultz et al., 2007; Hu and Yang, 2012). This animal model offers a unique opportunity to study the pathogenesis of *Mtb* and HIV/*Mtb* coinfection, as well as to determine how HIV alters protective CMI to *Mtb* in the context of BCG vaccination. These studies would greatly enhance progress toward understanding the mechanisms whereby HIV suppresses CMI to *Mtb* and accelerate design and screening of TB vaccines for HIV+ populations.

## Concluding remarks

Over the past two decades of intensive research, a myriad of TB vaccine candidates and adjuvants have been tested. Unfortunately, thus far, no efficacious vaccine has yet emerged. To our view, the greatest challenge in TB vaccine development is the understanding of the mechanisms by which *Mtb* evades and escapes the host innate and adaptive immune responses. Therefore, it is critical to continue basic research investigations aimed to fully elucidate immune evasion strategies used by *Mtb*. Knowledge gained from these studies will undoubtedly aid in the design of more rational vaccine approaches that will induce the immune system to overcome the anti-host defenses launched by *Mtb*, including drug resistant strains. We envision that the successful vaccine(s) will include, perhaps novel antigens, and importantly, vaccine delivery systems coupled to unconventional routes of immunization (e.g., nasal) that stimulate a yet to be unraveled type of immune response that is protective against a pathogen that has co-evolved with its hosts for thousands of years.

## Acknowledgements

Financial support: This work was supported by the following grants from the National Institutes of Health: R01 AI076425 to A. Campos-Neto.

### Conflict of interest statement

The authors declare that the research was conducted in the absence of any commercial or financial relationships that could be construed as a potential conflict of interest.
